# “Falling in and climbing out”: a qualitative study on vicarious trauma among hospice nurses

**DOI:** 10.1186/s12912-025-03845-9

**Published:** 2025-10-23

**Authors:** Yanming Wu, Yangchenchen Liu, Enhui Bo, Yuxin Zhou, Erming Yang, Ya Mao, Xingyue He, Yuanyuan Jin, Hui Yang, Huiling Li

**Affiliations:** 1 School of Nursing, Medical College of Soochow University, No. 1 Shizi Street, Suzhou, Jiangsu 215000 China; 2 Nursing College of Shanxi Medical University, Taiyuan, Shanxi China; 3 Department of Psychological Sciences, William & Mary, Williamsburg, VA USA; 4 Department of Psychology, University of Minnesota, Minneapolis, MN USA; 5 Department of Nursing, Zhongda Hospital Southeast University, Nanjing, Jiangsu China; 6 Cicely Saunders Institute of Palliative Care, Policy and Rehabilitation, Florence Nightingale, Faculty of Nursing Midwifery & Palliative Care, King’s College London, London, UK; 7 First Hospital of Shanxi Medical University, No. 55 Xinjian South Street, Taiyuan, Shanxi 030000 China

**Keywords:** Hospice nurses, Vicarious trauma, Qualitative study, Reflexive thematic analysis

## Abstract

**Background:**

Hospice nurses may encounter vicarious trauma during the empathy process with patients. However, vicarious trauma is a neglected issue in most hospice clinical nursing settings. The study aims to explore vicarious trauma experience and perspectives among hospice nurses.

**Methods:**

We conducted participatory observations in three hospitals and held semi-structured in-depth interviews with 16 hospice nurses from 9 cities between May and October 2023. Field notes and interview transcripts were analyzed as one coherent text by using reflexive thematic analysis.

**Results:**

Three main themes were identified: (1) “Falling in,” with subthemes of suffering from vicarious trauma and self-perceiving vicarious trauma; (2) “Struggling,” with subthemes of dealing with ambivalence; (3) “Climbing out,” with subthemes of “digesting” vicarious trauma and calling for organizational support.

**Conclusion:**

The findings indicated that hospice nurses are deeply affected by vicarious trauma. Nursing managers should pay more attention to the potential impacts of vicarious trauma on hospice nurses, and explore scientifically informed training courses and empowerment strategies to prevent and intervene in the vicarious trauma experiences of hospice nurses.

**Supplementary Information:**

The online version contains supplementary material available at 10.1186/s12912-025-03845-9.

## Key points

Hospice nurses are deeply affected by vicarious trauma and lack a clear understandings of it. In the absence of organizational support, they rely primarily on personal coping strategies and inner resilience to manage with the negative impacts of vicarious trauma.

Nursing managers should be attentive to the risk of vicarious trauma among hospice nurses and actively foster an open and supportive working environment that enables the timely identification of, and intervention for, nurses experiencing such trauma.

**Supplementary Information:**

The online version contains supplementary material available at 10.1186/s12912-025-03845-9.

## Background

Given the rapidly expanding older population and the rising prevalence of chronic illnesses, the demand for hospice care is increasing at an unprecedented pace [1, 2]. Patients often face severe physical illness that pushes them to the edge of death who experience not only physical pain from illness and symptoms but also complex psychological distress; therefore, they need more psychical and psychological assessments and care. Hospice care is a humanistic care service aimed at improving the quality of life for patients during their final stages, while also supporting family members through their grief [3, 4]. It provides specialized end-of-life support across various care settings, including in-patient settings, outpatient settings, and home settings [1]. Hospice nurses play a central role in hospice care, providing comprehensive support that addresses the physical, psychological, social, and spiritual needs of terminally ill patients. They deliver compassionate care that upholds the dignity of patients at the end of life [5].

Among all healthcare providers, hospice nurses work most closely with both patients and their families. On a journey where death is the inevitable outcome, hospice nurses often adopt an approach of “treating patients as family,” providing care that goes far beyond addressing physical needs [6]. They dedicate themselves both physically and emotionally, employing psychological support techniques such as empathy, perspective-taking, emotional connections, and personalized care [7, 8]. However, the ongoing responsibility of providing emotional support can take a toll on hospice nurses’ own well-being [9–11]. Through repeated and often unconscious exposure to traumatic environments involving patients’ suffering and families’ grief, their cognitive schemas may become disrupted. When empathic engagement becomes excessive, it can lead to the development of vicarious trauma (VT).

VT is defined as a psychological trauma that affects nurses’ cognitive schemas. It can arise in clinical settings or through exposure on social media, resulting from deeply empathizing with the physical or emotional trauma of patients, family members, or colleagues [12–15]. While VT and secondary traumatic stress (STS) are often used interchangeably [16], they represent distinct phenomena. VT involves a transformative process in which an individual’s cognitive schemas and beliefs are altered due to prolonged exposure to the trauma of others [12–15]. In contrast, STS is characterized by the outward symptoms similar to post-traumatic stress disorder (PTSD) symptoms, such as intrusive thoughts, hyperarousal, and avoidance behaviors, as a result of indirect exposures to traumatic events [17].

VT naturally arises from the interactions between nurses and patients during their caregiving and is considered an unavoidable occupational trauma for nurses [18]. Previous studies indicated that nurses across various specialties including psychiatric nurses [19], emergency nurses [20], and correctional nurses [21] experience VT to varying degrees. Hospice nurses, in particular, are chronically exposed to the trauma associated with patients’ deaths and are continuously emotionally invested in their daily care activities [22, 23]. Their frequent and profound empathy toward patients places them at a higher risk of experiencing VT compared to nurses in other settings. Musili and colleagues found that hospice care professionals are particularly vulnerable to VT due to their constant involvement in end-of-life care [24]. VT can result in both physical and psychological distress, and over time, it may alter nurses’ worldviews and core beliefs, ultimately impairing their ability to provide emotional support to patients with life-threatening illnesses [25]. This not only compromises the quality of care but also increases the psychological burden on nurses themselves [12]. VT has been identified as a significant contributor to job burnout among nurses [26, 27]. Burnout is known to be prevalent among hospice nurses [26–30]. However, the potential role of VT in contributing to the high rates of burnout among hospice nurses remains unexplored.

Given the influence of contextual factors, such as traditional Chinese cultural values of filial piety and prevailing views on life and death [31, 32], it is important to explore the unique experience of VT among hospice nurses within the Chinese cultural context [33]. Filial piety, a cornerstone of Confucian ethics, emphasizes respect for, care of, and responsibility toward one’s aging parents, including obligations related to end-of-life care [34, 35]. Traditional Chinese perspectives on life and death are deeply intertwined with filial piety, shaping attitudes toward caregiving, death, and bereavement [36, 37]. These cultural values may influence how hospice nurses perceive and experience VT, particularly in their emotional engagement and interactions with patients and families.

To date, only one qualitative study has explored VT among psychiatric nurses [19], highlighting a significant gap in the literature concerning hospice nurses. This underscores the urgent need to explore VT experiences specifically among hospice nurses and to develop appropriate empowerment strategies for this population. The aim of this study was to explore VT experience and perspectives among hospice nurses.

## Method

### Study design

This study was part of a larger project aimed at developing the measurement and intervention for VT among hospice nurses in China. This study used a descriptive-interpretive approach to explore VT experience and perspectives among hospice nurses. Descriptive-interpretive qualitative research is particularly rich in analyzing data at both the descriptive (surface) and interpretive (deeper) levels and telling a coherent story that weaves in historical context [38]. The COREQ checklist was followed to promote complete and transparent reporting of the study [39] (see Appendix S1).

### Sample

Due to the relatively small and geographically dispersed hospice nursing workforce in China, participants were recruited with purposive and snowballing sampling strategies. A study invitation letter (See Appendix S2) was provided to potential participants, which included the definition of VT and a description of its potential symptoms as outlined by McCann and Pearlman [13]. Potential participants were then asked to self-identify if they had experienced VT.

To be eligible for participation, hospice nurses had to meet the following criteria: (1) completion of professional hospice care training and more than one year of hospice care experience; (2) self-identification of having experienced or currently experiencing VT in their hospice care work; and (3) provision of informed consent to participate in the study. The exclusion criteria were: (1) nurses not currently working in hospice care or who had left hospice care roles; and (2) standardized training nurses, refresher nurses and nurse interns. Data collection continued until data saturation was reached, i.e., no new information emerged from subsequent interviews [40]. In total, 16 hospice nurses from nine institutions across multiple cities were included in this study.

### Data collection

This study used participant observations and semi-structured interviews to collect data. Participant observations involved researchers collecting data through direct engagement in practical activities alongside participants [41]. This method allowed the researchers to uncover insights that participants may not explicitly articulate or may be unaware of themselves [42]. Semi-structured interviews captured participants’ reflections on their personal experiences [43]. The integration of these two methods enabled the collection of rich, comprehensive, and valid data, allowing for an in-depth exploration of VT among hospice nurses [44].

#### Participant observation

We chose three hospice units located in Taiyuan, Suzhou, and Changsha through purposive sampling. After obtaining informed consent from the unit leaders, the first author spent two weeks at each site in the role of a “hospice trainee nurse” for the purpose of participate observation. The researcher shadowed hospice nurses during their daily routines, observing the dynamic interactions between patients and nurses, while continuously reflecting on the phenomenon of VT. The field notes included both descriptive entries detailing events in the field and reflective notes, which captured the researcher’s ideas, sensations, and interpretations. Field notes also encompassed brief excerpts from informal interviews conducted during observations, as these conversations were not audio-recorded.

#### Semi-structured interviews

Hospice nurses were interviewed individually and face-to-face by the first author in private rooms during field observations. Semi-structured interviews were guided by an interview protocol, which included questions such as: “What are your perspectives on vicarious trauma?”, “Have you experienced any traumatic events in your workplace?”, and “How did you overcome the traumatic events?” Follow-up questions were asked to encourage deeper reflection, particularly when participants shared specific stories related to VT. Each interview lasted about 60 min. All interviews were audio recorded.

### Data analysis

The field notes and interview transcripts were transcribed verbatim and analyzed as a single coherent dataset. Data was coded and analyzed using Braun and Clarke’s reflexive thematic analysis [45, 46]. The first and second authors independently read through the data to immerse themselves in the context. All transcripts were double coded using Microsoft Word. The authors met regularly to review the coded transcripts, discuss discrepancies, and reach consensus through interactive discussions. Initial codes were generated, and descriptive data segments were assigned. From these segments, the authors initiated the structuring process to identify potential themes. Through reflexive collaboration, the themes were reviewed and refined during team meetings. Finalized themes, subthemes, and categories were then shared with all team members to ensure they comprehensively captured hospice nurses’ experience of VT. Feedback from the team was incorporated, and the two authors further refined the themes in light of this input.

In practicing reflexivity, we contemplated our positionalities and how our personal and professional experiences might influence the research process and our interpretations. All authors possessed professional backgrounds in nursing or psychology, ranging from undergraduate to doctoral levels, which may have shaped pre-existing perspectives on VT. We remained aware of these influences throughout data collection and analysis, engaging in ongoing reflexive dialogue to ensure analytical rigor and interpretive integrity.

### Rigor

The first and second authors independently coded the data and then presented their findings for review and discussion with the broader research team. The validation process included questioning, challenging assumptions, and reflecting on personal and professional experiences. Additionally, the first author conducted member-checking by returning the study findings to participants to verify their accuracy and ensure that the interpretations resonated with participants’ lived experiences.

### Ethical considerations

This study was conducted in accordance with the ethical standards of the Declaration of Helsinki [47].Ethical approval was obtained from the Ethics Committee of the First Hospital of Shanxi Medical University in 2023 (Approval number: KYLL-2023-021). Participants were provided with detailed information about the study aim and interview procedures. Written informed consent was obtained from participants who were comfortable with documentation. For those who preferred not to sign forms due to cultural or personal reasons, oral informed consent was secured following IRB-approved protocols and documented by the researcher. Confidentiality was protected, and identifiable information was securely stored and de-identified. Participants were explicitly informed of their right to withdraw from the study at any time without retribution.

All interviews were conducted under the supervision of an academic advisor to ensure participant well-being, including providing immediate support if nurses experienced emotional distress. If such distress occurred, the interviewer would pause the session, provide emotional support, and offer a break. Participants were reminded of their right to withdraw or discontinue the interview if they felt overwhelmed. During the study, no participants reported excessive emotional distress or requested to terminate the interview. To minimize the risk of coercion, the interviewer had no personal or close relationships with participants. Participants were assured that their decision to participate or withdraw would have no impact on their professional or personal relationships.

## Results

**Fig. 1:**
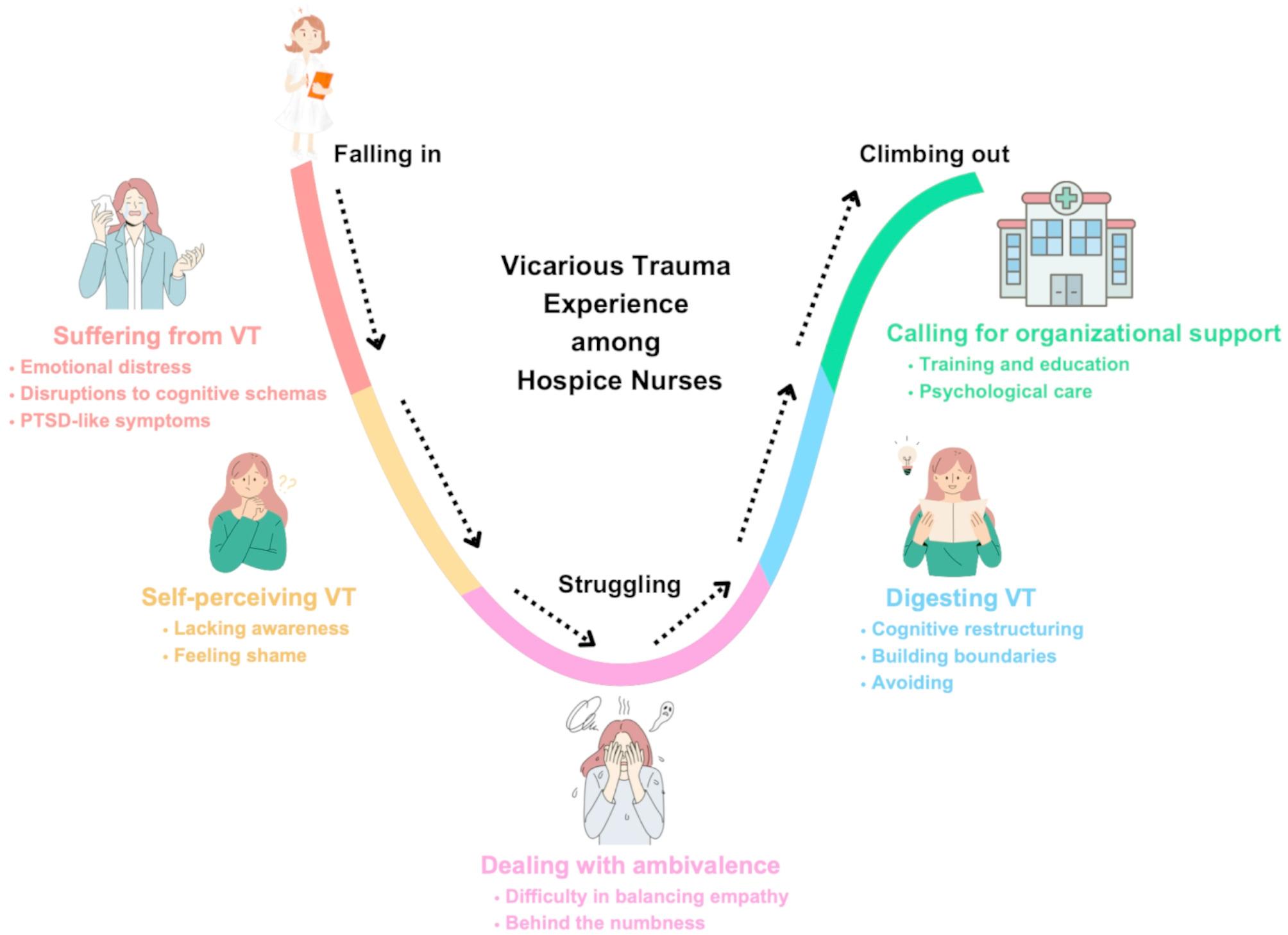
A visual representation of the results

All participants were identified as females and held higher education degrees. Fourteen of them held a bachelor’s degree (87.5%), one held an associate’s degree (6.25%), and one held a master’s degree (6.25%). Participants reported a diverse range of experience in delivering hospice care, ranging from one year to 15 years, with an average of four years of experience. The sample included individuals in various professional roles: twelve participants (75%) were registered nurses, three (18.75%) were senior registered nurses, and one (6.25%) was an associate chief nurse. Participants’ experiences and perceptions towards VT fell into three main themes and five subthemes (Fig. 1). Details on themes and quotes can be found in Table 1.

**Table 1:** Key themes and exemplar quotations

Overarching Themes	Themes	Sub-themes	Quotations
“Falling in”	Suffering from vicarious trauma	Shock of emotional distress	Just like a friend and this friend will leave soon, but there is no way to keep them alive. You just feel powerless. I want to help them to solve some problems to maintain their lives, but there just not solutions and just see them slowly disappear from me, from this world… (N3). My tears welled up as I spoke. I just think it’s quite meaningless. No matter how dedicated we are, even if I put all my time and efforts to this work, we won’t get any positive feedback of patients’ physical illness. No matter how well I perform and how strong my lumbar puncture technique is, they are still slowly dying. It’s like I hopelessly watch them approaching death every day. (N16). After working here for a long time, I always think about negative things or have negative feelings whenever any of my family members feels uncomfortable. I become extremely worried about the possibility of my family members developing a severe illness similar to that of my patients. (N6) I have suspected everyone in my family regarding their health and illness at some point. If anyone at home feels discomfort in their stomach, I immediately become worried and afraid, thinking they might have a tumor. My family doesn’t understand my worries and anxieties; they think I’m always suspicious and too neurotic. However, since I face these illnesses every day, I just can’t help but feel that the possibility of my family members getting sick is very high. (N8).
		Disruptions to cognitive schemas	In the last moments of life, no one in a good mood. The individuals I interact with every day are pessimistic emotions and I need to confront gloomy circumstances. After witnessing these life-and-death situations, I sometimes feel losing hope to my life. I behave become very pessimistic. I feel that life is just like that way, and everything seems meaningless. (N11).
			Sometimes I would place myself in my patients’ situations and experience their emotions. For instance, if the patient is a senior, I may relate to or picture my future of growing old and potentially being treated with indifference by my children. That’s heart-breaking. How can a child doesn’t take care of their parents? It goes against our traditional values and beliefs—very different. It has shattered my worldviews. (N14).
		PTSD-like symptoms	After the patient passed away, I would sometimes experience flashbacks while driving. I would abruptly stop at red lights, feeling scared. It has brought me tremendous shocks and severely impacted my life. The images of working with the patients, our conversations, they just kept flashing back in my head again and again… (N7). I always cry with no reasons; I just want to cry after facing those negative energy day by day. For example, I suddenly felt a strong wave of sadness and upset while measuring the blood pressure of a senior patient. There was no specific reason for it; I just kept crying and crying… (N11). Do you know the word? Nightmares—they happen very often. I consistently dream about the dying moments of my patients. Senior patients have remarked that we always send them on their way to death, and I think it gives nightmares. (N16).
	Self-perceiving vicarious trauma	Lacking awareness	You unconsciously fall into patients’ worlds and remain stuck in empathy even outside of work. It’s been a long time for me of being trapped in the whirlpool of emotions filled with memories and sorrows, and it’s hard to break free. I was aware that I became overly emotional and empathetic, but I had no solution. I didn’t know what vicarious trauma was; I just endured it and suffered from it for a long time. (N3).
			Vicarious trauma is a strange term for me; I didn’t really notice its existence. We often discuss burnout and compassion fatigue, but nobody talks about establishing boundaries and being cautious about the presence of vicarious trauma. I’m the kind of person who can easily become overly involved, always getting too close to my patients in the workplace. Vicarious trauma absolutely is a problem that needs to be addressed. (N9).
		Feeling shame	I am the kind of person who can easily relate to and become involved in others’ emotions, even though I recognize it may not be entirely appropriate or professional. I sometimes deny and question my professionalism. As a professional nurse, one is expected to be mentally strong and avoid becoming overly involving in patients’ emotions. I feel a sense of shame about it and perceive myself as immature. (N7). As a professional, I should always maintain an image of sanity. Vicarious trauma makes me appear unprofessional, and it is challenging to discuss with others. (N4).
“Struggling”	Dealing with ambivalence	Difficulty in balancing	If you’re not emotionally involved with your patients, they won’t trust you or openly share their feelings. However, you shouldn’t empathize too much; I have found no solution for balancing it most of the time. (N13). I can let those emotions go nowadays, and it releases me from distress. But becoming overly empathetic is unavoidable, and I find it hard to balance… It just swings back and forth. I feel lost sometimes. (N5).
		Behind the numb	I think I shouldn’t build an emotional connection with patients anymore. If I give in too much emotional empathy, it would burn out my energy, and I just started working like a robot without emotion, becoming cold and numb. It’s meaningless to provide hospice care in that way. It’s not me, and I won’t be happy at work. Therefore, I must contribute emotional connection with my patients again, even if I experience emotional distress and burnout. (N16).
“Climbing out”	“Digesting” vicarious trauma	Cognitive restructuring	At the beginning of the work, I felt upset every time. Later, I gradually changed my cognition. For example, when my patient passed away, I imagined them going to heaven and becoming a baby there. I told myself, “Everything happens inevitably. Don’t lock yourself up and huddle in a corner. I need to move on in life.” (N1). Field notes: After today’s interview, nurse N14 shared a passage with me describing their cognitive restructuring that occurred after an awareness of over-empathy: “I’ve always been the kind of person who believes in ‘burning myself to light up others.’ After reflecting on that period of experience, I shouldn’t be a ‘candle-type’ person but rather a ‘flashlight-type’ person—illuminating the way when needed but able to be turned off and rest easily, so I can take care of myself.” (N14).
		Building boundaries	I used to dedicate all my efforts and time wholeheartedly to my patients, but I felt too hurt. I also found it hard to get out of those emotions, so now I hold back a bit. I just focus on my work and do what I’m supposed to do. There is still emotional involvement, but I make sure to maintain boundaries in it. (N9) I try to maintain a balance and offer more professional advice to my patients from a nurse perspective instead of engaging in personal emotion from a personal perspective. I can actively listen to their complaints and pains, but I won’t become overly involved in their experience and emotions. It’s crucial to build boundaries. (N16).
		Avoiding	I know she (a patient) would never come back, and it saddens me. I transferred all the photos of her from my phone to my home laptop and sealed them in a hidden corner. I rarely looked at them, just hid them away because I couldn’t bear the emotions after seeing those picture or thinking of her. (N2) You can help them within your capacity and ability, but you can’t involve empathy with them. Once you have empathetic engagement, it’s extremely hard for you to get out of those emotions, and it brings hurts to your feelings. I would never involve empathy with them. (N12). Field notes: Another terminal patient passed away and nurse N3 felt upset and helpless. She shared her feelings with me: “I really hope that one day, I won’t have to bear too much emotional involvement, but if I don’t involve emotion, you simply cannot walk into their world and connect with them. If there are better options of working environment, I could choose to leave.” (N3).
	Calling for organizational support	Training and Education	I think that we need training courses to instruct and guide us in properly coping with vicarious trauma. For instance, theoretical knowledge should be delivered during college which help us to practically use them in clinical works. It also provides mental preparation in the future. Therefore, I can identify if I have issues as well as I know how to regulate and further solve them. (N7). I really think that we need training on how to build boundaries and get out of those feelings and situations after connecting to others’ emotions. (N10).
		Psychological care	I don’t want to get involved in overwhelming emotions anymore, as they bring a sense of helplessness from which no one can rescue me. I truly hope that someone can provide us with some support and care, helping us navigate through these emotions. (N10). I cried for the entire night after the patient passed away. Many times, I feel that we experience even more sorrow than the patients’ families. I think we also need grief counseling. (N16).

### “Falling in”

Hospice nurses regularly and directly confront patients’ pains and deaths, and they are expected to provide patients with emotional support. In the course of this caregiving, they inevitably engage in deep—sometimes excessive—empathy, which can lead to negative psychological consequences. Hospice nurses described VT as an experience of “falling in.”

#### Suffering from vicarious trauma

During the process of “falling in,” hospice nurses reported various levels of emotional distress, disruptions to cognitive schemas, and even PTSD-like symptoms.

(1) Emotional distress

When providing hospice care to terminally ill patients, nurses were responsible not only for delivering end-of-life comfort measures and managing physical symptoms, but also for navigating emotionally complex situations in which they felt “unable to provide” or “could not provide” further care. These moments often placed them in deeply distressing and frustrating circumstances. Many nurses reported intense feelings of powerlessness and guilt when they perceived that there was nothing more they could offer to support their patients.

*Just like a friend and this friend will leave soon*,* but there is no way to keep them alive. You just feel powerless. I want to help them to solve some problems to maintain their lives*,* but there just no solutions and just see them slowly disappear from me*,* from this world…* (N3).

In the hospice care department, nurses work with terminally ill patients who exhibit various physical symptoms. Prolonged exposure to an environment dominated by illness and death led many nurses to develop heightened anxiety about the health and safety of their own family members.

*After working here for a long time*,* I always think about negative things or have negative feelings whenever any of my family members feels uncomfortable. I become extremely worried about the possibility of my family members developing a severe illness similar to that of my patients.* (N6)

(2) Disruptions to cognitive schemas

Disruptions to cognitive schemas is a core characteristic of VT. VT can significantly disturb hospice nurses’ worldviews and belief systems, though the extent varies among individuals. Prolonged exposure to death-related trauma—combined with the emotional suffering of patients and their families—gradually contributes to the development of increasingly negative perspectives on life and care. In the context of Chinese culture, which places strong emphasis on achieving a peaceful and fulfilling death, additional strain arises when nurses witness the absence of children at a parent’s deathbed. This conflict between the cultural ideal of filial piety and the reality of unmet expectations can profoundly undermine nurses’ beliefs about the fulfillment of filial duties. Among hospice nurses, the cognitive disruptions caused by VT are most notably expressed through pessimistic and shattered worldviews.

*In the final moments of life*,* no one is in a good mood. The people I interact with every day are filled with pessimism*,* and I’m constantly surrounded by gloomy circumstances. After witnessing so many life-and-death situations*,* I sometimes feel like I’m losing hope in my own life. I become very pessimistic. It feels as if this is all life amounts to—everything starts to seem meaningless.* (N11)

(3) Suffering from PTSD-like symptoms

VT poses significant risks to nurses’ mental health, especially when it becomes severe or remains unaddressed. Nurses reported experiencing PTSD-like symptoms, including flashbacks, uncontrollable crying, and nightmares:

*After the patient passed away*,* I would sometimes experience flashbacks while driving. I would abruptly stop at red lights*,* feeling scared. It has brought me tremendous shocks and severely impacted my life. The images of working with the patients*,* our conversations*,* they just kept flashing back in my head again and again…* (N7)

#### Self-perceiving VT

VT is a common occupational hazard arising from nurses’ empathic engagement with patients and their families. As such, developing an accurate understanding and awareness of VT is essential for its prevention. However, many hospice nurses remain unfamiliar with the concept and often perceive it as a sign of personal weakness or lack of professionalism, which may lead to feelings of stigma. This lack of proper understanding hinders their ability to recognize the early signs of VT, leaving them vulnerable to its cumulative effects. As a result, some nurses experience the shock of VT without appropriate strategies to manage it, gradually becoming more deeply affected by its psychological impact.

(1) Lacking awareness.

Many hospice nurses reported that the concept of VT was vague, and this ambiguity and confusion hindered them from applying appropriate strategies to prevent and cope with VT.

*You unconsciously fall into patients’ worlds and remain stuck in empathy even outside of work. It’s been a long time for me of being trapped in the whirlpool of emotions filled with memories and sorrows*,* and it’s hard to break free. I was aware that I became overly emotional and empathic*,* but I had no solution. I didn’t know what vicarious trauma was; I just endured it and suffered from it for a long time.* (N3)

(2) Feeling shame

Nurses often questioned their own professionalism and emotional resilience when they found themselves in tears as a result of deep empathic engagement or the cumulative effects of VT. Some even experienced a profound sense of shame in response to these emotional reactions.

*I am the kind of person who can easily relate to and become involved in other’ emotions*,* even though I recognize it may not be entirely appropriate or professional. I sometimes deny and question my professionalism. As a professional nurse*,* one is expected to be mentally strong and avoid becoming overly involving in patients’ emotions. I feel a sense of shame about it and perceive myself as immature.* (N7)

### Struggling

Nurses often find themselves in a state of internal struggle after “falling in” but before they are able to employ coping strategies to “climb out.” This phase represents a deeply personal and mentally exhausting process of navigating emotional distress.

#### Dealing with ambivalence

This form of struggle often manifests as a deep sense of ambivalence, particularly around how much empathy to extend to patients. Hospice nurses frequently wrestled with the challenge of offering appropriate levels of empathic engagement while protecting their own emotional well-being.

(1) Difficulty in balancing empathy

Hospice nurses emphasized that empathy is essential to building trust and providing meaningful hospice care. However, excessive empathy often led to emotional distress, leaving them mentally drained and unsure of how to maintain boundaries. One nurse reflected:

*If you’re not emotionally involved with your patients*,* they won’t trust you or open up. But if you empathize too much*,* it really takes a toll. Most of the time*,* I haven’t found a way to strike the right balance.* (N13)

(2) Behind the numbness

Many hospice nurses described experiencing emotional numbness as a manifestation of VT. However, this numbness did not signify indifference. Rather, it reflected a prolonged buffering process, during which nurses continued to invest emotionally in their patients and families despite ongoing emotional distress. This repeated cycle—offering empathy, experiencing emotional distress, and returning to empathetic care—left nurses in a constant state of internal struggle, grappling with the cumulative impact of VT. The numbness, in this context, emerged as a protective response to the emotional toll of sustained empathic engagement.

*I started thinking that I shouldn’t build emotional connections with patients anymore. When I give too much emotionally*,* it drains me completely—I burn out. At one point*,* I was working like a robot*,* emotionless*,* cold*,* and numb. But providing hospice care that way felt meaningless. It wasn’t me*,* and I wasn’t happy in my work. So even though it brings emotional distress and burnout*,* I know I have to reconnect emotionally with my patients. That’s the only way the care feels real—and the only way I feel like myself.* (N16)

### “Climbing out”

Hospice nurses described coping with VT as a “climbing-out process.” The metaphor of “climbing” captures the strenuous and deliberate nature of this effort—requiring the use of one’s whole body, every available resource, and sustained emotional energy, all while contending with fear and vulnerability.

#### “Digesting” vicarious trauma

Hospice nurses described that after experiencing the emotional shocks of VT, they primarily relied on individual efforts to buffer and cope with its negative impacts.

(1) Cognitive restructuring

Many hospice nurses expressed a strong desire to bring comfort and relief to their patients, often pushing themselves beyond their personal and professional limits. After confronting the emotional toll of VT, they gradually came to recognize the limitations of what they could realistically achieve in improving patient outcomes. Through cognitive restructuring, nurses began to reframe their expectations—actively accepting these limitations to avoid excessive empathy and emotional burnout. By consciously adjusting their mindset, they sought to strike a healthier balance between compassionate engagement and self-care, thereby reducing the long-term psychological impact of VT.

*Field notes: After today’s interview*,* nurse N14 shared a passage with me describing their cognitive restructuring that occurred after an awareness of over-empathy: “I’ve always been the kind of person who believes in ‘burning myself to light up others.’ But after reflecting on that period*,* I realized I shouldn’t be a ‘candle-type’ person. Instead*,* I want to be more like a flashlight—someone who can provide light when needed but can also be turned off to rest*,* so I can take care of myself.”* (N14)

(2) Building boundaries

Hospice nurses believed that VT was a manifestation of “crossing boundaries.” Therefore, nurses expressed the need to learn to balance empathic engagement and build boundaries in nurse-patient relationships to prevent VT and cope with the negative effects.

*I used to dedicate all my time and effort wholeheartedly to my patients*,* but I ended up feeling deeply hurt. I found it difficult to escape those emotions*,* so now I hold back a little. I focus on my work and do what I’m supposed to do. There is still emotional involvement*,* but I make sure to maintain clear boundaries.* (N9)

(3) Avoiding

Hospice nurses also employed a series of avoidance strategies after experiencing a tremendous empathic crisis and VT. These strategies included “sealing memories in hidden corners,” actively reducing or discontinuing emotional involvement, and, in extreme cases, leaving the position to mitigate the potential negative effects of VT. Three nurses shared their experiences of using avoiding strategies but in different ways:

*I know she (a patient) would never come back*,* and it saddens me. I transferred all her photos from my phone to my home laptop and sealed them away in a hidden corner. I rarely looked at them*,* just hid them because I can’t bear the emotions that come from seeing those picture or thinking of her.* (N2)

*You can help them within your capacity and ability*,* but you can’t get emotionally involved with them. Once you engage empathically*,* it’s extremely hard to detach from those emotions*,* and it hurts. I choose never to involve empathy with them.* (N12)

*Field notes: Another terminal patient passed away and nurse N3 felt upset and helpless. She shared her feelings with me: “I really hope that one day*,* I won’t have to bear too much emotional involvement*,* but if I don’t involve emotion*,* you simply cannot walk into their world and connect with them. If there were better work environments*,* I might consider leaving.”* (N3)

#### Calling for organizational support

Hospice nurses recognized that “Digesting’ vicarious trauma” of VT is a skill that requires conscious, repeated practice and training to effectively prevent and mitigate its impact. As such, organizational involvement is crucial in facilitating this process. However, many nurses reported relying primarily on self-regulated coping strategies and often felt overwhelmed by their limited ability to manage VT effectively. They expressed a strong desire for their organizations to establish structured support systems—such as VT-informed training programs and accessible mental health resources—to enhance their capacity to prevent and cope with VT.

##### (1) Training and education

Nurses emphasized that training and education are essential for raising awareness of VT and for developing appropriate coping strategies. They recommended more systematic training in affective empathy and trauma-informed care, which would help them understand the potential risks of VT and enhance their ability to recognize, manage, and mitigate its impact.

*I think that we need training courses to instruct and guide us in properly coping with vicarious trauma. For instance*,* theoretical knowledge should be delivered during college which help us to practically use them in clinical works. It also provides mental preparation in the future. Therefore*,* I can identify if I have issues as well as I know how to regulate and further solve them.* (N7)

##### (2) Psychological care

Hospice nurses reported being emotionally impacted by VT, particularly in the context of their highly emotionally demanding work environment. This emotional toll affected their motivation to continue providing hospice care. Many nurses expressed a strong desire for access to psychological support services to help them cope with the negative effects of VT.

*I don’t want to be overwhelmed by emotions anymore—they bring a deep sense of helplessness that no one can pull me out of. I sincerely hope that someone can offer us support and care*,* to help us navigate through these emotions.* (N10)

## Discussion

This study found that hospice nurses are deeply affected by VT and lack a clear understandings of it. In the absence of organizational support, they rely primarily on personal coping strategies and inner resilience to manage with the negative impacts of VT. A key contribution of this study is the development of a conceptual framework (see Fig. 1) that conceptualizes VT as a dynamic and evolving process shaped by individual, relational, and contextual factors. This framework suggests that VT can be addressed though targeted support and intervention. It offers a foundation for future research on VT across diverse clinical and cultural contexts and informs the development of targeted strategies to support hospice nurses in managing VT.

Hospice nurses described their experience of VT as a feeling of “falling in.” Emotionally, participants frequently reported two predominant forms of distress: powerlessness and guilt. This could be attributed to the inevitability of death in severe illnesses, causing medical professionals to experience a sense of powerlessness and guilt, conflicting with their career values rooted in the responsibility of “healing people and saving lives” [48]. It also reflects the conflict between the expectations of traditional medical profession to saving lives and the concepts of hospice care [49]. In terms of cognition, many hospice nurses reported that their fundamental worldviews were challenged during the process of emotionally interacting with dying patients, aligning with the disruption of cognitive schemas in VT [25, 50]. Hospice nurses believed that the helplessness arising from the realization that “No matter what care I provide, their deaths are unavoidable” forces them to experience a tremendous sense of meaninglessness, especially after long-term work in an environment full of negative emotions, facing the pessimism and helplessness of patients, thus gradually leading to a pessimistic worldview.

Additionally, “shattered worldviews” is identified as another consequence, mentioned by the nurses, resulting from disruptions to cognitive schemas. It mainly refers to the dismantling of traditional filial piety views held by hospice nurses [31]. In traditional Chinese moral standards, “filial piety is the most important virtues”, and filial piety is placed on a uniquely important position in families, personal interactions, and the society [51]. The traditional filial piety view has become the standard for people’s behavior, such as providing sufficient both physical and psychological companionship for the dying elderly [52]. Therefore, the refusal of some families of terminal patients to accompany and provide end-of-life care has greatly challenged nurses’ values and left them in significant self-doubt. The experiences of trauma severely disrupt and challenge ingrained belief systems, leading to internal dissonance and psychological distress [53]. Last, most participants described experiences of PTSD-like symptoms such as flashbacks and nightmares. This finding is consistent with previous studies that when VT is severe and not addressed with appropriate and effective care, healthcare providers’ mental health can be affected, resulting in PTSD symptoms, depression, and other mental health problems [54–56].

The current study’s results indicated that hospice nurses are unfamiliar with the concept of VT as well as feel shame about it. Lacking awareness of VT and feeling shame about experiencing it prevent nurses from adopting appropriate preventive and coping strategies to buffer the negative effects of VT. This can lead hospice nurses to “fall deeper and deeper” unconsciously and worsen their experience of “falling in.” Previous studies suggest that a lack of recognition or denial may lead to misdiagnosis, prolonging the impact of VT [57]. The current study found that many hospice nurses have minimal background knowledge regarding the concept of VT, and they lack the ability to articulate their experience of VT even when undergoing severe physical and mental conditions. Even if hospice nurses recognize their emotional distress or physical discomfort and try to attribute it to causes such as high work stress, lack of rest, or poor physical health, they are still unable to convince themselves or fail to fully adjust the conditions. During the process of hospice care, hospice nurses must employ empathy to build trusting relationships with patients for providing higher quality care. Empathy is the “best assistant” for hospice nurses in delivering care; however, it also functions as an “assassin”, posing a threat to their health and increasing the potential risk of experiencing VT during deep empathic engagement. Therefore, it is necessary for hospice nurses to be fully informed and aware of the underlying risks of VT. Pearlman pointed out that self-awareness is the starting point of recovering from the symptoms of VT [14]. Additionally, understanding one’s own needs and appropriately adjusting physical and mental states can help prevent and cope with VT. According to the United States Substance Abuse and Mental Health Services Administration (SAMHSA), trauma-informed care includes four R’s: realize, recognize, response, and resist re-traumatization. Therefore, nurses should fully understand fundamental trauma-informed care [58], consistently engage in self-awareness and reflection (e.g., encourage journaling or storytelling practices to help nurses process difficult experiences and reclaim purpose), and continuously build psychological strength and professional confidence to mitigate the impacts of VT to the greatest extent (e.g., offer resilience training grounded in compassion satisfaction). Importantly, VT is a naturally occurring occupational risk based on human nature. It is normal to accept one’s behaviors and reactions, and it should not be perceived as abnormal or unprofessional with a shameful or blaming attitude toward oneself [59, 60]. McCann also indicated that helpers’ feelings of security and safety in the workforce need to be ensured, rather than being negatively affected or even shamed for asking for help [13]. Therefore, normalizing the phenomenon of VT and alleviating the stress associated with its stigmatization could help hospice nurses self-protect appropriately and effectively.

We also found that hospice nurses are unable to actively or successfully cope with VT. Due to their prolonged exposure to death-related trauma and continuous empathetic engagement with patients’ emotions, hospice nurses undergo repeated struggles. Hospice nurses struggle to find a balance between empathic engagement and maintaining boundaries, and this challenging situation leads them to experience numbness to varying degrees. In fact, the experience of numbness emerges as a self-protective strategy adopted by hospice nurses to prevent excessive empathy. This finding is consistent with Lipsky’s study [61], which revealed that helpers often experience emotional numbness when they are unable to empathize with others’ situations; the reason for this numbness is their inability to cope with and process the constant and overwhelming nature of events. On the other hand, our findings suggest that behind the numbness, hospice nurses, through self-reflection, strive to strike a balance by incorporating an appropriate amount of empathy to prevent VT during their empathy with terminal patients. During the process of “struggling,” hospice nurses gradually become more active and positive in finding potential solutions to cope with VT, which lay a foundation for further “climbing out.”

In this study, hospice nurses described their hardship and struggles in coping with VT as a process of “climbing out.” We found that hospice nurses cope with VT through cognitive restructuring, building boundaries, and other strategies. Cognitive restructuring helps individuals recognize and change irrational mindsets, eliminate negative thoughts and views, and establish healthy and rational beliefs, which is consistent with previous studies. Moreover, hospice nurses applied the strategy of building emotional boundaries and establishing an appropriate distance to prevent falling into VT due to blurred boundaries. Similar to Kim et al.’s study findings, nurses should establish appropriate boundaries and maintain an emotional distance with patients to avoid experiencing VT from frequent empathic engagement [62]. Skovholt also suggested that the ideal state of empathy is for nurses to understand and involve themselves in patients’ worlds; at the same time, nurses need to be cautious about self-differentiation and learn the essential skills of maintaining emotional distances [63]. From the moment nurses begin engaging with patients’ traumatic experiences, the core of the dynamic process through which VT accumulates lies in how individuals undertake, adjust, differentiate, and integrate these experiences. Additionally, some hospice nurses indicated that when constantly experiencing the struggles of empathic trauma and feeling the unbearable negative impacts of VT, they often choose avoidance strategies such as reducing or stopping empathic engagement with patients, and even quitting the hospice care position. This also confirms what has mentioned in the literature: VT can reduce nurses’ empathy, making them insensitive to the physical and mental suffering of patients, lowering their motivation for work, affecting the quality and safety of nursing services, and even leading to a tendency to resign, threatening the stability of the nursing team [19, 25, 64].

Organizations and institutions hold essential responsibility in preventing and managing the phenomenon of VT [65]. However, this study found that organizations have not formed well-structured supportive systems in helping hospice nurses prevent and cope with the potential risks of VT. In most of the cases, nurses just and only could rely on their own skills and abilities in coping with VT. More and more studies point out that organizations should pay close attention to this issue and take their responsibility to develop right attitudes and specific supportive methods to help staff effectively mitigate the issue of VT [66, 67]. In our study, hospice nurses are unfamiliar with the concept of VT and lack the correct perception of it, which, from certain aspects, also indicates the insufficiency of institutional training and education. It is worth noticing that hospice nurses have indeed learned many skills in providing hospice care to patients during their fundamental education and daily training. However, they have rarely been taught effective and appropriate methods for caring for themselves [68–70]. Education and training are the key to raising awareness of VT and designing relevant interventions. Nursing organizations have the responsibility to educate nurses about the potential risks and assist them in recognizing and coping with their experience of VT. When nurses anticipate potential occupational risks, they maintain awareness of their experiences and feelings, thus becoming willing to spend time identifying the impacts resulted from caring for patients. Therefore, we believe that, in addition to training their skills of actively listening and engaging empathy during the hospice care training process, nurses need to be fully informed about the importance of building emotional boundaries. The improvement of professional skills will empower nurses to better handle individual cases, establish reasonable expectations of caring outcomes, and reduce the possibility of experiencing VT among hospice nurses. Additionally, most studies have found that essential psychological supports bring benefits in coping with VT [71]. Accordingly, hospice care managers should be attentive to the risk of VT that hospice nurses may face during the caregiving process. They should also actively foster an open and supportive working atmosphere so that nurses who are experiencing VT can be promptly identified and intervened when needed.

## Implication

The findings of this study have significant implications for hospice nursing practice. It is crucial to incorporate information about the experiences of VT and its impacts on hospice nurses’ sense of self and empathic responsiveness into hospice nurse training programs. Such training can better prepare nurses for trauma-informed care by increasing awareness of the emotional effects of working with dying patients. In addition, supervisors and administrators play a critical role in supporting hospice nurses by fostering a psychologically safe and supportive work environment. This includes providing ongoing education about the emotional toll of end-of-life care, diversifying caseloads to prevent cumulative exposure to trauma, and encouraging engagement in activities that promote emotional resilience—such as personal counseling, meditation, and journaling.

## Strengths and limitations

This study has several strengths. First, the use of a qualitative study design enabled an in-depth exploration of hospice nurses’ experiences of VT, providing rich and nuanced insights into an understudied topic. Second, the research team’s combined skills and expertise in nursing and psychology contributed to the depth of the data analysis. Third, methodological rigor was enhanced through independent coding procedures and regular team discussions, which helped to reduce potential bias. Finally, the study’s attention to the cultural contexts of China adds to the growing body of literature on VT in non-Western settings [13].

This study however has several limitations. First, it may not adequately capture individual differences in how VT is perceived and experienced, such as the influence of gender and personal trauma histories. Second, the researchers’ professional backgrounds in nursing and psychology may have introduced pre-existing assumptions or biases. To mitigate this, we engaged in reflexive practices throughout the research process. Additionally, while purposive and snowball sampling were appropriate given the relatively small and dispersed population, these methods carry an inherent risk of selection bias. The study’ s focus on VT also introduced potential ethical concerns, particularly around emotional vulnerability and power imbalances. To address this, participants were fully informed of their right to withdraw from the study without consequence. Future studies can consider incorporating more diverse perspectives, including male nurses from different cultural backgrounds, to further validate and expand upon the findings of this study.

## Conclusion

This study represents an important extension of existing research. Based on our findings, it is essential that healthcare leaders and educators recognize the potential impacts of VT on hospice nurses and prioritize the development of relevant training programs and empowerment strategies. While the principle of “patient-centered” care remains vital, it is equally important to attend to the voices of hospice nurses. They should be provided with structured support, including guidance on emotional control, stress management strategies, and the use of psychological resources.

## Supplementary Information

Below is the link to the electronic supplementary material.

Supplementary Material 1

## Data Availability

All the raw data (including participants’ voice files and the texts of the interviews) will be confidential and will not be able to share publicly. However, the codes that emerged during the current study are available from the corresponding author upon reasonable request.
