# Supplementary material for: “Falling in and climbing out”: a qualitative study on vicarious trauma among hospice nurses
Source: BMC Nurs. 2025 Oct 23;24:1320. doi: 10.1186/s12912-025-03845-9 (PMC12551335; doi:10.1186/s12912-025-03845-9)
Supplement: Supplementary file 1 — Supplementary Material 1 [file 12912_2025_3845_MOESM1_ESM.docx]

**Supplementary Files**

Appendix S1. Research reporting checklist (COREQ Checklist)

Appendix S2. Research invitation letter

**Appendix S1. Research reporting checklist (COREQ Checklist)**

| **Section and Topic** | **Item #** | **Checklist item** | **Location where item is reported** |
| --- | --- | --- | --- |
| **DOMAIN 1: RESEARCH TEAM AND REFLEXIVITY** | | |  |
| **Personal Characteristics** | | |  |
| Interviewer/facilitator | 1 | Which author/s conducted the interview or focus group ? | Page 7 |
| Credentials | 2 | What were the researcher’s credentials ? E.g. PhD, MD. | Title page |
| Occupation | 3 | What was their occupation at the time of the study ? | Title page |
| Gender | 4 | Was the researcher male or female ? | Title page |
| Experience and training | 5 | What experience or training did the researcher have ? | Page 8 |
| **Relationship with participants** | | |  |
| Relationship established | 6 | Was a relationship established prior to study commencement ? | Page 7 |
| Participant knowledge of the interviewer | 7 | What did the participants know about the researcher ? e.g. personal goals, reasons for doing the research. | Page 7 |
| Interviewer characteristics | 8 | What characteristics were reported about the interviewer/facilitator? e.g. Bias, assumptions, reasons and interests in the research topic. | Page 8-9 |

| **Section and Topic** | **Item #** | **Checklist item** | **Location where item is reported** |
| --- | --- | --- | --- |
| **DOMAIN 2: STUDY DESIGN** | | |  |
| **Theoretical framework** | | |  |
| Methodological orientation and Theory | 9 | What methodological orientation was stated to underpin the study ? e.g. grounded theory, discourse analysis, ethnography, phenomenology, content analysis. | Page 5-6 |
| **Participant selection** | | |  |
| Sampling | 10 | How were participants selected ? e.g. purposive, convenience, consecutive, snowball. | Page 6 |
| Method of approach | 11 | How were participants approached ? e.g. face-to-face, telephone, mail, email. | Page 7-8 |
| Sample size | 12 | How many participants were in the study ? | Page 6 |
| Non-participation | 13 | How many people refused to participate or dropped out ? Reasons ? | Page6 |
| **Setting** | | | |
| Setting of data collection | 14 | Where was the data collected ? e.g. home, clinic, workplace. | Page 7-8 |
| Presence of non-participants | 15 | Was anyone else present besides the participants and researchers? | Page 7-8 |
| Description of sample | 16 | What are the important characteristics of the sample ? e.g. demographic data, date. | Page 6 |
| **Data collection** | | | |
| Interview guide | 17 | Were questions, prompts, guides provided by the authors ? Was it pilot tested ? | Page 8 |
| Repeat interviews | 18 | Were repeat interviews carried out? If yes, how many ? | Page 8 |
| Audio/visual recording | 19 | Did the research use audio or visual recording to collect the data ? | Page 8 |
| Field notes | 20 | Were field notes made during and/or after the interview or focus group ? | Page 7 |
| Duration | 21 | What was the duration of the interviews or focus group ? | Page 8 |
| Data saturation | 22 | Was data saturation discussed ? | Page 6 |
| Transcripts returned | 23 | Were transcripts returned to participants for comment and/or correction ? | Page 9 |
| **DOMAIN 3: ANALYSIS AND FINDINGSZ** | | | |
| **Data analysis** | | | |
| Number of data coders | 24 | How many data coders coded the data ? | Page 8 |
| Description of the coding tree | 25 | Did authors provide a description of the coding tree ? | Page 9 |
| Derivation of themes | 26 | Were themes identified in advance or derived from the data ? | Page 8 |
| Software | 27 | What software, if applicable, was used to manage the data ? | N/A |
| Participant checking | 28 | Did participants provide feedback on the findings ? | Page 9 |
| **Reporting** | | | |
| Quotations presented | 29 | Were participant quotations presented to illustrate the themes / findings ? Was each quotation identified ? e.g. participant number | Page 10-17 |
| Data and findings consistent | 30 | Was there consistency between the data presented and the findings ? | Page 10-17 |
| Clarity of major themes | 31 | Were major themes clearly presented in the findings ? | Page 10-17 |
| Clarity of minor themes | 32 | Is there a description of diverse cases or discussion of minor themes ? | Page 10-17 |

**Appendix S2. Research invitation letter**

Dear Prospective Participant:

Welcome!

My name is XXX, and I am a nursing doctoral student at the Nursing College of XXX University. I am conducting a qualitative study on vicarious trauma among hospice nurses.

Vicarious trauma refers to negative changes in nurses' view of self, others, and the world, resulting from empathetic engagement with traumatized individuals. Hospice nurses are chronically exposed to the trauma of death and are continuously emotionally invested in daily nursing activities. Their frequent and deep empathy toward patients may put them in a higher risk of experiencing vicarious trauma compared to nurses in other units. However, vicarious trauma is a neglected issue in hospice nurses. Therefore, this study aims to use qualitative research to explore gaps in the literature focused on the experiences and perspectives of hospice nurses with vicarious trauma. Based on the literature review and clinical observations, I have summarized the symptoms of vicarious trauma, which specifically include the following aspects: ①Physiological issues: nightmares, insomnia, decreased appetite, accelerated heart rate, chest discomfort, headache, and so on. ②Psychological problems: Negative emotions like guilt, depression, fear, anger, and numbness. Constant work-related worries and excessive involvement with patients lead to experiencing past traumas or patients' described traumatic scenes and sensations. Decreased sensitivity to personal needs distorts nurses' worldviews, affecting their trust, safety, control, self-esteem, and intimacy. Distance from family and friends due to fear of being misunderstood or disliked. Doubts about nursing abilities and contemplating leaving the profession.

Having read the description about vicarious trauma, if you: ① Received certification from professional hospice care training and obtained more than one year of hospice care experience. ②Feeling that you have encountered some of the symptoms I mentioned while working and recognizing that you have experienced vicarious trauma. ③Willing to share your life story to help other nurses with similar experiences. I sincerely invite you to join me in exploring the essence and journey of vicarious trauma, listening to your life story, and reflecting on the impact of this experience and how it intertwines with your other life experiences.

If you agree to share your life story, this study would require your assistance as follows: ①I will conduct 1-2 in-depth interviews with you, each lasting about an hour. You can stop the interviews at any time if you feel uncomfortable; I will completely respect your decision. ②The entire interview will be recorded using a recording device for accuracy. Recorded data will be confidential and deleted after the study. You can stop the recording anytime or request that specific content not be used in the research. ③The tapes will be transcribed into written transcripts, and I will send them to you for review and reflection. Please feel free to provide any corrections or feedback.

I sincerely invite you once again to participate in this study. Your involvement will contribute to the improvement of hospice nurses' psychological well-being and quality of professional life. If you are willing to participate or have any questions about this research, please feel free to contact me!

Sincerely,

XXX

Phone number: XXX

E-mail: XXX
